# Revitalization of the Waterfront Park Based on Industrial Heritage Using Post-Occupancy Evaluation—A Case Study of Shanghai (China)

**DOI:** 10.3390/ijerph19159107

**Published:** 2022-07-26

**Authors:** Qiao Zhang, Jooho Lee, Bin Jiang, Gunwoo Kim

**Affiliations:** 1 Graduate School of Urban Studies, Hanyang University, 222 Wangsimni-ro, Seongdong-gu, Seoul 04763, Korea; nwfa98@163.com (Q.Z.); coascape@hanyang.ac.kr (J.L.); 2 Urban Environments and Human Health Lab, HKUrbanLabs, Faculty of Architecture, The University of Hong Kong, Hong Kong SAR, China; jiangbin@hku.hk; 3 Division of Landscape Architecture, Department of Architecture, The University of Hong Kong, Hong Kong SAR, China

**Keywords:** industrial site, urban revitalization, Houtan Park, Xuhui Binjiang Park, user experience

## Abstract

With the rapid development of urban construction, the waterfront industrial heritage park has played an active role in shaping the city’s image, regional economic development and environmental improvement, and the continuation of the city’s waterfront history. The waterfront park based on industrial heritage using post-occupancy evaluation will help improve the sustainable management, maintenance, and design level of the project in the future. However, there is insufficient research on the waterfront industrial heritage park using post-occupancy evaluation. This paper takes Shanghai Houtan Park and Xuhui Binjiang Park, the representative industrial heritage parks in China, as the research objects. Through field investigation and nearly 200 questionnaires and interviews regarding user behavior, the importance of design elements (place characteristics, natural environmental characteristics, usability characteristics, and administrative characteristics) and the correlation of satisfaction help us to understand the use of the two parks and analyze and organize the survey data, carrying out the analysis of the questionnaire results using frequency analysis, IPA analysis, *t*-test, variance analysis, and multiple regression analysis. The research results include: (1) Both parks are mainly used by people in their twenties and forties, and the trail received the highest utilization rate as the main facility, while the visitor center in charge of guiding functions had the lowest utilization rate. (2) While Houtan Park received high satisfaction with natural environmental characteristics, it was found that Xu Hui Binjiang Park had relatively high satisfaction with the place and usability characteristics. (3) The natural environmental characteristics of Houtan Park have a positive impact on overall satisfaction and return visit satisfaction. Site characteristics and utilization characteristics of Xuhui Binjiang Industrial Park have a positive impact on overall satisfaction, while usability characteristics have a positive impact on return visit satisfaction. Finally, according to the questions and suggestions raised by users, an optimization strategy is proposed for the renewal of the park, and it is hoped that it can provide suggestions for the reconstruction and design of similar Chinese waterfront industrial heritage parks.

## 1. Introduction

Most of the world’s major cities have formed around rivers. However, as China’s industry changed and the economy developed, most traditional sectors located in the city’s waterfront area either disappeared or were neglected. Instead of merely considering these sectors as empty spaces in an abandoned town, we intend to recognize them as a new type of urban resource and a starting point for reinventing the city.

Industrial heritage includes the factories, warehouses, logistics transportation facilities, and infrastructure where the operation was suspended and the original functions remained lost. After the 1980s, the world’s leading cities began studying ways to utilize the several industrial facilities that were built during the Industrial Revolution. Since 1990, some cities in the West have highlighted historical and regional values and identified potentials areas for the aforementioned purpose [1]. The developed industrial heritage recreational space can promote the employment of surrounding residents, attract population backflow, slow down the decline in urban functions, bring direct social benefits, and at the same time drive the appreciation of surrounding assets [2]. In particular, industrial heritage-based parks were created by introducing natural ecological elements to improve the environment. These sites are being treated more importantly because they affect local revitalization beyond the functions of existing parks [3].

After the reform and opening of the 1980s, China neglected numerous traditional industries in the city center as industrial restructuring and technological innovation began in earnest. By 2000, several industrial heritage sites of historical and cultural significance were demolished, and skyscrapers were built instead. The National Cultural Heritage Administration announced the “Wuxi Proposal” charter in June 2006 and began recycling industrial heritage. According to a 2014 National Statistical Office survey, 74 percent of Chinese residents cited the creation of parks as the most efficient recycling method for underdeveloped industrial areas [4].

Several cities in China are currently developing—or at least planning to develop—industrial heritage-based parks. The increasing public interest has stimulated the active creation of industrial heritage parks. However, most cases fell short of the expected effect, and there was a lack of identity, civic participation, and continuous management [4].

Since post-occupancy evaluation plays an essential role in the management and improvement of industrial heritage parks, studying appropriate measures through post-occupancy evaluation is necessary to further revitalize the industrial heritage park.

The history of Chinese industrialization begins with the Opium Wars of 1840. Shanghai, the birthplace of modern Chinese industry [5], has grown into a significant industrial hub since China’s reform and opening up. Currently, Shanghai has the highest level of urbanization and industrialization in China. After the port opened in 1843, Shanghai had several factories along the Huangpu River. The Huangpu River, with a total length of 113 km, existed as a source of economic activity in Shanghai. The banks of the Huangpu River have always been an important development axis of Shanghai and a symbolic space of the city. However, the industrial area located along the Huangpu River declined due to the transformation of Shanghai’s economic development method in the 20th century. As a result, most industrial facilities, such as docks and factories, either disappeared or were neglected.

In the early 21st century, Shanghai emphasized the development of waterfront space centered on the Huangpu River, striving to build the Huangpu River into a golden waterfront that highlights the core competitiveness of Shanghai and a world-class waterfront area with international influence. Many famous cities in the world are located at the intersection of large rivers or sea and land. The convenient water transportation conditions not only facilitate the daily operation of the city, but also often make the multicultural collision and fusion here, forming a unique charm [6]. The Huangpu River in Shanghai is the spatial carrier of the core functions of a global city, and a gathering place for financial trade, cultural creativity, scientific and technological innovation, and R&D functions with global influence. It is an urban public living room with rich humanistic connotations, and a symbolic display window that reflects high-level cultural influence and humanistic vitality. The development and reuse of the waterfront space of the Huangpu River is one of the important ways to integrate and enhance urban functions. By improving the quality of waterfront space, it can also effectively promote the development of regional economy, reshape the image of the city, and demonstrate the soft power of Shanghai. To this end, the Shanghai People’s Government has promoted the Huangpu River Comprehensive Development Project since 2002. The waterfront park promotion project was an essential aspect of the comprehensive development project of the Huangpu River and an essential means to regenerate the industrial zone along the Huangpu River. The waterfront park was not only used to solve urban problems but also formed the foundation of new life within the city. Waterfront parks have been promoted as new waterfront green leisure spaces, as people’s desire for enhanced quality of life has increased [7].

This study aims to: Firstly, analyze the relevant theories of the waterfront industrial heritage park and sort out the design elements of the waterfront industrial heritage park; Secondly, take Shanghai Houtan Park and Xuhui Binjiang Park as the research objects, analyze the design elements affecting these two parks according to the post-occupancy evaluation, and verify the impact path; Finally, make recommendations for development and improvement. Shanghai Houtan Park and Xuhui Riverside Park, as representative cases of China’s waterfront industrial heritage parks, have certain reference and reference value for the development of China’s developing waterfront industrial heritage parks.

## 2. Materials

There is not enough research on the analysis of post-occupancy evaluations of industrial heritage parks. Among them, the research content of post-use evaluation of industrial heritage is not different from the post-occupancy evaluation research of general parks, and only analyzes the frequency of users’ usage patterns and facility usage satisfaction. In order to solve these problems, this study improves the deficiencies that are present in previous research and aims to carry out the following tasks: (1) Through the research and analysis of the waterfront industrial heritage park at home and abroad, the problems and design methods of the park are summarized. The development status of China’s waterfront industrial heritage parks is reviewed. (2) This paper addresses the design elements of the waterfront industrial heritage park through literature research and then analyzes the relevant evaluation indicators. (3) Using frequency analysis, IPA analysis, *t*-test, variance analysis, and multiple regression analysis, this paper analyzes the results of the questionnaire.

### 2.1. Cases of Waterfront Industrial Heritage Parks

The aforementioned cases are summarized as follows. Rivers brought about the formation and transformation of urban forms and had an irreplaceable effect on the development of urban social economy. Large cities in Europe, the United States, and the East focused on the development and use of waterfront areas. Among them, a project to park a waterfront site with many industrial heritage resources was making a lot of progress. Waterfront parks based on industrial heritage are a new type of urban waterfront green area, which means that they can be actively used to promote the construction of urban parks and rest systems while balancing the urban waterfront ecological environment. In the case of the West, most industrialization has been completed, and accordingly, the design and practice of waterfront parks based on industrial heritage were relatively large. In the West, the design techniques are relatively mature, so it was judged to be an example that can be referred to in the design of waterfront parks based on industrial heritage in oriental cities. In addition, it can be seen that many countries in the West do not achieve protection and recycling of industrial heritage alone by the government, but through the cooperation of various organizations. Support for the protection or recycling of industrial heritage can be only provided when certain funds are guaranteed. Although the degree of protection and utilization of industrial heritage varies depending on the original functional conditions of the waterfront site, it was found that there were many similar parts to the design technique. In the case of the East, the development project of the waterfront area has reached the stage of rapid progress, but there are not as many designs of waterfront parks based on industrial heritage as in the West. In addition, the introduced design techniques are relatively uniform and have problems in terms of maintenance, but it can be seen that they are paying more attention to improving the environment of the waterfront area. Currently, among eastern countries, China, in particular, lacks overall awareness of the redevelopment of large waterfront industrial heritage sites. It was judged that it was necessary to refer to the Western rational industrial heritage recycling ideology in the city’s overall plan, and it was thought that it was necessary to avoid damaging the historical aspect or identity of industrial heritage when establishing regional plans (Table 1).

### 2.2. The Development and Current Situation of China’s Industrial Heritage Parks

Since ancient times, China has used Ze Shui Erju (choose water to live in) and Iin Shui Jiancheng (build a castle near the water) as basic principles for constructing cities because it has influenced the Feng Shui theory of Beishan Linshui (back to the mountain, adjacent to the water) in many waterfront cities. Waterfront cities account for 67.65% of China’s capital cities. As the traditional Chinese manufacturing industry waned, closed quarries, factories, train stations, ports, and waste landfills became urban blind spots, damaging urban landscapes and damaging residents’ lives [25].

To solve such problems in the early 21st century, China began to turn industrial heritage into parks. Qijiang Park, Zhongshan City, Guangdong Province, was completed in 2000 and is China’s first waterfront park utilizing industrial heritage. After its opening in China, the government and citizens became increasingly aware of the importance of the industrial heritage park. Citizens have begun to realize that by transforming industrial heritage into parks, it can not only arouse people’s memories of places and life, but it can also improve the regional ecological environment and meet people’s needs for outdoor recreation and place perception.

With the 2010 Shanghai World Expo, many industrial heritages were regenerated in the city center. In particular, industrial heritages along the Huangpu River appear to the public as examples of parks. Therefore, according to the industrial culture development promotion policy announced by the Ministry of Industry and Information Technology and the Ministry of Finance of China in January 2017, industrial heritage-based parks should be actively developed as essential resources of industrial culture. Since then, the local government has been actively establishing plans for industrial heritage parks.

Industrial heritage-based waterfront parks are one of the effective ways to regenerate and develop urban waterfronts. With the support of the city and local governments, more and more waterfront industrial heritage-based park projects are being implemented in these cities. Waterfront parks based on industrial heritage are mainly located in waterfront cities such as Shanghai, Wuhan, Gwangju, and Nanjing, where economic development is fast and post-industrial society has been in progress for a long time [26]. The Hu Industrial Park in China restores the ecological environment of the site through scientific and artistic recycling of facilities such as natural elements, structures, buildings, and machinery in an abandoned industrial district located in the city and creates a new green landscape [19]. It is said to be a suitable place for citizens to create and engage in activities. Gao [27] mentioned that it is an open space with industrial characteristics that can improve the ecological environment of a place and provide rest and experience to urban residents by remodeling, recycling, and combining ecological design techniques. Under the premise of respecting its history in the city, restoring the site through landscape design and ecological design creates a green industrial heritage park.

### 2.3. Evaluation Index of Waterfront Parks Based on Industrial Heritage

The general characteristics of waterfront parks based on industrial heritage differ somewhat depending on the research purpose in the existing literature and research on the industrial heritage park in the waterfront area. Focusing on the research contents, it is largely divided into four types: ‘place characteristics’, ‘natural environmental characteristics’, ‘usability characteristics’, and ‘management characteristics’. The design elements included in each type of characteristic are summarized in Table 2.

After removing conceptually overlapping contents, the synthesis results and reorganization of analysis indicators are shown in Table 3.

## 3. Methods

### 3.1. Study Area

Shanghai is the most developed area in China for industrial heritage protection and recycling business. Shanghai’s industrial heritage is mainly distributed along the coasts of the Huangpu River and Woosong River [38]. The Shanghai People’s Government has promoted the Hwangpo River Comprehensive Development Project since 2002. In particular, the development of experience tourism resources with Shanghai industrial and cultural characteristics was emphasized by utilizing existing waste industrial buildings, docks, and various structures along the river. Therefore, the promotion of the industrial heritage waterfront park is an important part of the Hwangpo River industrial experience tourism development project and an important means of regenerating the industrial heritage area along the Hwangpo River. In particular, with the 2010 Shanghai World Expo, industrial heritage was recycled along the Hwangpo River. Among them, many urban public spaces that recycle industrial heritage into waterfront parks not only improve the quality of citizens’ lives, but also form a new landmark along the Huangpu River in Shanghai. It meets the theme of the 2010 Shanghai Expo, Beautiful City, Happy Life. Shanghai Houtan Park and Xuhui Binjiang Park were built along the Huangpu River during that period, making them Shanghai’s first industrial heritage parks.

**Figure 1:**
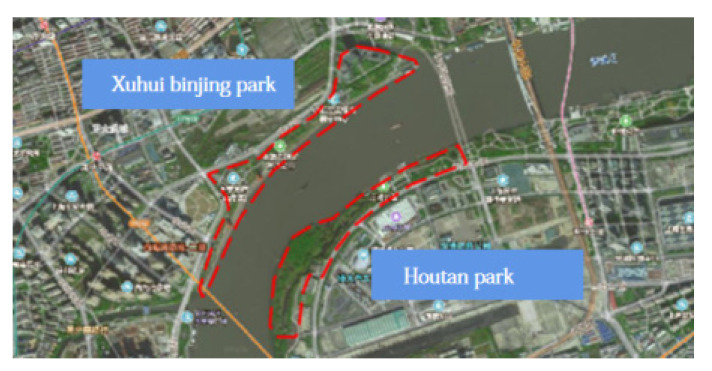
Spatial scope of the study, 2011. Source: Google Maps.

Moreover, Shanghai Houtan Park and Xuhui Binjiang Park are examples of China’s industrial heritage-based parks, which are worthy of study and have a pilot effect nationwide in Figure 1. Additionally, Shanghai Houtan Park and Xuhui Binjiang Park are similar in terms of the construction period, relocation site characteristics, and area. In contrast, the two parks exhibit differences in how the industrial heritage and operational management are used. Therefore, for this study, Shanghai Houtan Park and Xuhui Binjiang Park were considered worthy of comparison and thus selected as the study sites (Table 4).

### 3.2. Survey Questions and Survey Method

To assess users’ general status, the research questionnaire comprised questions regarding sex, age, occupation, education, and residence. The usage behavior was composed of transportation means, arrival time, frequency of use, seasonal use, day of week of use, time of use, length of stay, visit companions, reason for visit, place mainly visited, and facility mainly used. Thereafter, questionnaire items were composed to analyze the importance and satisfaction of the post-occupancy evaluation of the industrial heritage-based park. Regarding the overall satisfaction level, items such as overall satisfaction, revisit intention, and recommendation intention were selected. The evaluation items of factors affecting satisfaction with industrial heritage-based parks comprised four items: location characteristics, natural environmental characteristics, usability characteristics, and management characteristics.

The respondents’ demographic characteristics and usage behavior characteristics are given a nominal scale to enable frequency analysis in the questionnaire setting.

A four-point Likert scale was used to investigate the importance, satisfaction, and overall satisfaction of a Chinese industrial heritage-based park according to its post-occupancy evaluation. The survey is summarized in Table 5.

The questionnaire was randomly distributed at the entrance of the park and filled out truthfully (including 44 multiple-choice questions), as well as relevant interviews about which areas of the park need to be improved. The preliminary survey is conducted to review the appropriateness of the questionnaire and guidance before conducting this survey. On Friday, 10 August 2018, 30 Shanghai Houtan Park users were selected for one day to review the problems that will arise in this survey in advance. Reliability analysis by Chronbach’s alpha was conducted for the preliminary survey results, and the detailed figures are as follows.

As shown in the above results, the reliability analysis results of the importance and satisfaction of the waterside park evaluation project based on industrial heritage show that all alpha values are calculated at a high value of 0.8 or more, and it can be seen that the difference between the design projects provides high internal consistency for measuring the same concept (Table 6).

After the preliminary survey, this survey, which was conducted after correcting and supplementing defects in the questionnaire, included weekdays and weekends for a week from 10–17 March 2019, and surveyed 100 copies of each of the two target sites by 9 a.m. As a result, a total of 198 questionnaires were collected and 195 valid samples were recovered as three parts of the questionnaire that caused errors. The basic information of park users and the degree of performance and importance of park-related elements and the use of facilities were obtained, in order to obtain more extensive research results.

### 3.3. Statistical Analysis Method

For the survey analysis, the SPSS 24.0 statistical program was used, and for the survey items, frequency analysis, reliability analysis, technical statistical analysis, IPA analysis, *t*-test, variance analysis, and multiple regression analysis are conducted.

(1) Through frequency analysis, the demographic characteristics and usage behaviors of survey respondents were analyzed.
(2) Reliability analysis was performed to determine the degree of internal consistency between park satisfaction and the importance of measurement variables. Thereafter, descriptive statistical analysis was performed to determine satisfaction and importance using the derived design elements as a five-point Likert scale.
(3) IPA analysis was conducted to reveal the priorities when evaluating and improving the achievement by region, analyzing the relationship between satisfaction and importance for the two parks’ design elements.
(4) The *t*-test and variance analysis are conducted to test whether there is a difference in satisfaction with design elements according to demographic characteristics and visit characteristics.
(5) Multiple regression analysis was performed to verify the relationship between independent satisfaction variables of the park design elements and user behavioral intentions.

## 4. Results

### 4.1. Analysis of Demographic Characteristics and Users’ Behavioral Characteristics

(1) Comparing the gender distribution of users of Houtan Park and Xuhui Binjiang Park, women showed a slightly higher composition ratio than men (Table 7).
(2) In the case of age, the users of the second target site were mainly in their twenties and forties, and those in their thirties were the main users. Users in their twenties used Houtan Park more, and users in their forties used Binjiang Xuhui Park more.
(3) In the case of occupations, office workers were surveyed at the highest rate in the two subjects analyzed.
(4) In the case of academic background, college graduates and masters occupy most of the two subjects, so it can be seen that most of them are highly educated.
(5) In the case of residence, users of Houtan Park mainly reside in Pudong-gu, east of the Huangpu River. On the other hand, it can be seen that citizens living in the west of the Huangpu River mainly use Xuhui Binjang Park. In addition, the number of visitors to Houtan Park is more than that of Xuhui Riverside Park.
(6) As for transportation, users of Houtan Park mainly used public transportation, and users of Xufu Binjang Park mainly walked.
(7) The arrival time was the highest rate for both parks by 10–30 min.
(8) Regarding the number of uses, it was found that Houtan Park users usually use it once every 2–3 days, and Xuhui Binjang Park users usually use it once a week, and it was found that Houtan Park was more popular than Xuhui Binjang Park for the first time.
(9) According to the analysis results of the usage season, day of use, and time of use, Houtan Park is often used in the afternoon regardless of the season, and Xuhui Binjang Park is used on weekends and evenings regardless of the season (Table 8).
(10) The largest number of users stayed at Houtan Park for 30 min to 1 h, and the largest number of users stayed at Xuhui Binjang Park for 1 h to 2 h.
(11) There are quite a few people who usually use Houtan Park with their families and co-workers, and many people usually use Xuhui Binjang Park with their families and alone.
(12) In terms of the reason for visit, there were many users who mainly took a walk, enjoyed looking at nature, and explored the project in Houtan Park, and it can be seen that users usually take a walk, exercise, and rest in Xuhui Binjang Park.
(13) Most of the places used for the two target sites were water-friendly areas. The facilities mainly used by users of Houtan Park were the most frequently used wetland trails, and users of Xuhui Binjang Park most frequently used waterfront trails (Table 9).
(14) The most satisfying point was that users of Houtan Park chose eco-friendly scenery, and users of Xuhui Binjang Park chose to preserve industrial heritage.
(15) The most unsatisfactory point was that users of Houtan Park mainly chose a lack of park commentary data, a lack of habitat information, and a lack of use programs as issues that must be improved, while users of Xuhui Binjang Park mainly chose a lack of facilities, a lack of park commentary data, and a lack of use programs (Table 10).

**Table 7:** Demographic characteristics of users by destination.

Park Name		Houtan Park		Xuhui Binjiang Park	
Type		Frequency	Percentage (%)	Frequency	Percentage (%)
Sex	Male	44	45.4	48	49.0
	Female	53	54.6	50	51.0
Age	Under 20	4	4.1	6	6.0
	20s	30	30.9	25	25.5
	30s	34	35.1	36	36.7
	40s	14	14.4	20	20.4
	50s	8	8.2	6	6.1
	Over 60	7	7.2	5	5.1
Job	Student	10	10.3	16	16.3
	Public employee	4	4.1	2	2.0
	Private employee	45	46.4	44	44.9
	Homemaker	4	4.1	5	5.1
	Self-employed	11	11.3	13	13.3
	Technician	9	9.3	7	7.1
	Professor	5	5.2	1	1.0
	Retired	9	9.3	7	7.1
	Others	-	-	3	3.1
Education level	Lower than high school	12	13.4	15	15.3
	High school graduated	13	12.4	15	15.3
	Bachelors	48	49.5	45	45.9
	Masters	22	22.7	20	20.4
	PhD	2	2.1	3	3.1
	Others	-	-	3	3.1
Residence	Pudong District	70	70.2	5	5.1
	Xuhui District	-	-	64	65.3
	Yangpo District	3	3.1	1	1
	Hongkou	1	1	-	-
	Jeongan District	1	1	-	-
	Huangpo District	1	1	13	13.3
	Putuo District	3	3.1	4	4.1
	Shanghai Suburban Area	1	1	6	6.1
	Outside Shanghai	17	17.5	5	5.1

**Table 8:** Characteristics of user usage behavior for destination 1.

Park Name		Houtan Park		Xuhui Binjiang Park	
Type		Frequency	Percentage (%)	Frequency	Percentage (%)
Transportation	By walking	25	25.8	33	33.7
	Public transportation (bus, subway, etc.)	29	29.9	20	20.4
	Private car	25	25.8	24	24.5
	Bicycle	11	11.3	14	14.3
	Taxi	7	7.2	7	7.1
	Others	-	-	-	-
	All previous	97	100	98	100
Arrival time	Less than 10 min	13	13.4	15	15.3
	10–30 min	52	53.6	51	52.0
	30 min–1 h	25	25.8	29	29.6
	1–2 h	5	5.2	3	3.1
	More than 2 h	2	2.1	-	-
	All previous	97	100	98	100
Frequency of use	Everyday	12	12.4	13	13.3
	Once every two or three days	22	22.7	19	19.4
	Once a week	19	19.6	25	25.5
	Once a month	8	8.2	15	15.3
	Once a season	7	7.2	9	9.2
	Once every six months	5	5.2	4	4.1
	Once a year	4	4.1	1	1.0
	First visit	20	20.6	12	12.2
	All previous	97	100	98	100
Seasonal use	Spring	33	34	19	19.4
	Summer	3	3.1	7	7.1
	Fall	8	8.2	7	7.1
	Winter	-	-	-	-
	Regardless of season	53	54.6	65	65
	All previous	97	100	98	100
Use by day	Weekdays	17	17.5	9	9.2
	Weekend	32	33.0	52	53.1
	Public holiday	3	3.1	5	5.1
	Regardless of the day	45	46.4	32	32.7
	All previous	97	100	98	100
Use by time	Morning	13	13.4	2	2.0
	Lunchtime	20	20.6	10	10.2
	Afternoon	39	40.2	29	29.6
	Dinner	3	3.1	38	38.8
	Regardless of the time	22	22.7	19	19.4
	All previous	97	100	98	100
Stay time	10–30 min	6	6.2	6	6.1
	30 min–1 h	39	40.2	28	28.6
	1–2 h	29	29.9	43	43.9
	More than 2 h	23	23.7	21	21.4
	All previous	97	100	98	100
Accompanying visit	Alone	25	25.8	24	24.5
	Friends	20	20.6	17	17.3
	Couple	11	11.3	8	8.2
	Family	26	26.8	44	44.9
	Colleagues	13	13.4	5	5.1
	Others	2	2.1	-	-
	All previous	97	100	98	100
Reason for visit (multiple answers)	Walk	42	34.4	45	36.3
	Exercise	11	9.0	31	25.0
	Rest	13	10.7	25	20.2
	Picnic	12	9.8	12	9.7
	Photography	10	8.2	3	2.4
	Attending an event or meeting	1	0.8	1	0.8
	Seeing/enjoying nature	18	14.8	2	1.6
	Date	3	2.5	-	-
	Natural ecological learning	-	-	-	-
	Other (project exploration)	12	9.8	5	4
	All previous	122	100	119	100
Most used place	Waterfront	71	73.2	65	66.3
	Binjiang activity zone	24	24.7	12	12.2
	Industrial heritage recycling zone	2	2.1	21	21.4
	All previous	97	100	98	100

**Table 9:** Characteristics of user usage behavior for destination 2.

Park Name		Houtan Park			Xuhui Binjiang Park	
Type		Frequency	Percentage (%)		Frequency	Percentage (%)
Facilities mainly used—multiple answers	Waterfront walkway	11	6.0	Waterfront walkway	76	39.4
	Abandoned factory garden	10	5.5	Skateboard square	33	17
	Tree square	6	3.3	Art museum	26	13.5
	Wetland walkway	74	40.4	Train and warehouse restaurant	-	-
	Running track	21	11.5	Binjiang plan exhibition space	2	1.0
	Observatory	42	23.0	Railroad garden	19	9.8
	Restaurant	4	2.2	History square	7	3.6
	Yacht wharf	4	2.2	Rock climbing space	27	14.0
	Visitor center	10	5.5	Wharf garden	3	1.6
	Drifting garden	1	0.5	Visitor center	-	-
	All previous	183	100	All previous	193	100

**Table 10:** Characteristics of user usage behavior for destination 3.

Park Name		Houtan Park		Xuhui Binjiang Park	
Type		Frequency	Percentage (%)	Frequency	Percentage (%)
Most satisfying part	Good management	5	5.2	5	5.1
	Program access	1	1.0	9	9.2
	Ease of access	2	2.1	7	7.1
	Preservation of industrial heritage	4	4.1	46	46.9
	Eco-friendly landscape	85	87.8	24	9.2
	Diversity of facilities	-	-	7	7.1
	Others	-	-	-	-
	All previous	97	100	98	100
Most dissatisfying part	Park management status	-	-		
	Insufficient program	16	16.5	17	17.3
	Difficult access	11	11.3	3	3.1
	Lack of safety at the waterfront	2	2.1	-	-
	Insufficient habitat information	24	24.7	7	7.1
	Lack of park commentary material	33	34.0	32	32.7
	Lack of facilities (e.g., toilets, drinking fountain, parking)	11	11.3	33	33.7
	Others	-	-		
	All previous	97	100	98	100

### 4.2. Reliability Analysis

In this study, the Cronbach’s alpha value of the item for the importance of the design elements of Houtan Park was 0.819, and the Cronbach’s alpha value of the item for the satisfaction was 0.809. Furthermore, the Cronbach’s alpha for the importance of the design elements of Xuhui Binjiang Park was 0.851, and the Cronbach’s alpha for satisfaction was 0.821. The Cronbach’s alpha is higher than 0.60, which is generally considered reliable in social sciences; therefore, the questionnaire on the importance and satisfaction of design elements in this study can be judged to have high reliability (Table 11).

### 4.3. Descriptive Statistical Analysis

Houtan Park visitors selected environmental regeneration in neglected and damaged areas as an essential factor in designing an industrial heritage park. Surface trace preservation and re-creation were selected as relatively unimportant elements of the design. Regarding satisfaction, the pleasant natural scenery was selected as the most satisfying industrial heritage park design element. The program was selected as the most unsatisfactory factor among the design elements for industrial heritage parks.

Visitors to Xuhui Binjang Park selected harmonious natural scenery as an essential factor in designing an industrial heritage park. Surface trace preservation and re-creation were selected as relatively unimportant elements of the design. Regarding satisfaction, maintenance was selected as the most satisfactory factor among the design elements of an industrial heritage park. However, it was selected as a minor satisfactory element among design elements such as programs and information facilities (Table 12).

### 4.4. IPA Analysis

**Figure 2:**
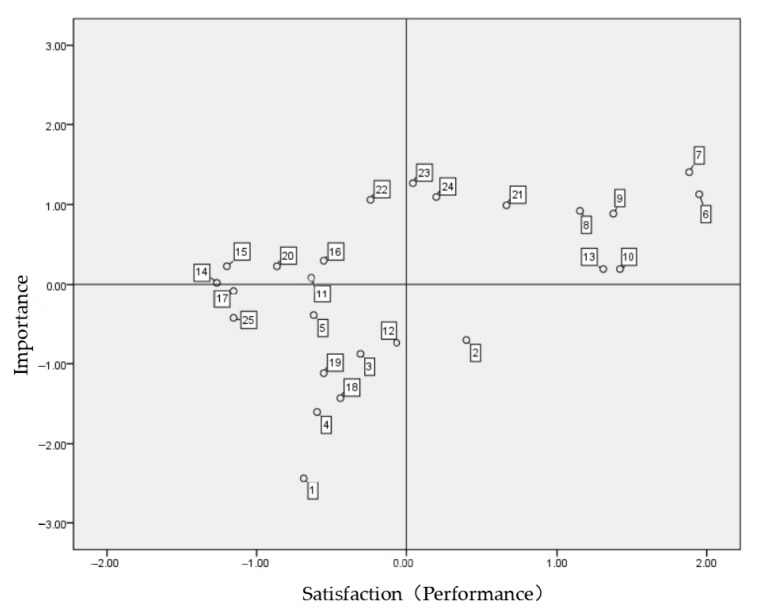
Houtan Park IPA analysis. Source: SPSS.

In Houtan Park, management characteristics—access, programs, information facilities, convenience facilities, lighting facilities, and safety—are essential. However, satisfaction is low; thus, improvements should be urgently implemented. In particular, the park has a safety problem due to the night lighting facility. Furthermore, the lowest use rate and lowest evening use satisfaction indicate that issues concerning lighting facilities and safety maintenance should be addressed immediately (Figure 2).

Additionally, items regarding the maintenance of cleanliness, facilities, and plant ecology for Houtan Park were located in the superiority maintenance area, indicating the need for better maintenance and revitalization. The importance and satisfaction of related items are relatively low due to the location characteristics of Houtan Park. Furthermore, most users were unaware that Houtan Park was used as an industrial heritage site.

**Figure 3:**
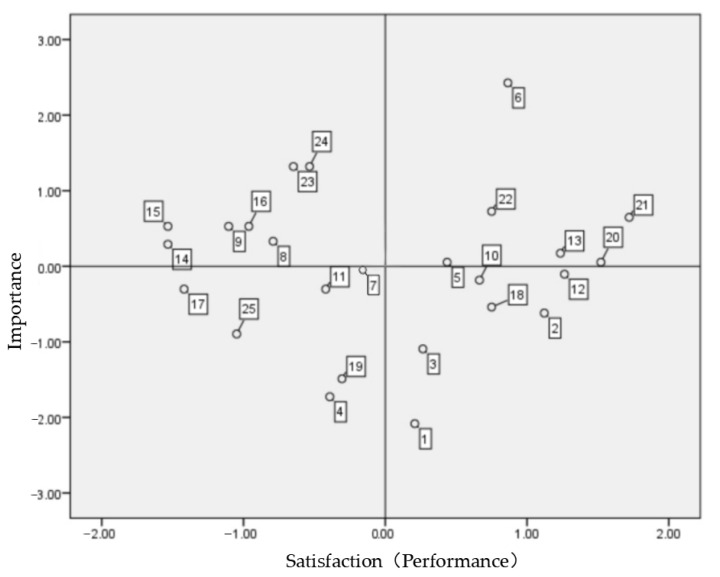
Xuhui Binjiang Park IPA analysis. Source: SPSS.

In Xuhui Binjang Park, the elements of “harmonious natural scenery,” “easy to access,” “sports facilities,” “lighting,” “cleanliness,” and “safety” are located in the general maintenance area; thus, better maintenance is needed in the future (Figure 3).

However, local history is reflected through the primary activities of the site characteristics, plant diversity from the natural environmental characteristics, programs from polluted water purification and utilization characteristics, information facilities, convenience facilities, and facility and plant maintenance. Ecological maintenance has emerged as a problem and needs improvement. Here, the most dissatisfying aspects from the analysis of user behavior are consistent with the lack of facilities for use, lack of commentary data on parks, and insufficient use of programs.

Additionally, it is necessary to recognize the importance of characteristic site elements—such as “preservation and re-creation of surface traces,” “preservation of symbolic facilities and buildings,” and “recycling of waste facilities into park facilities”—through active publicity (Table 13).

### 4.5. Analysis of Differences in Satisfaction with Design Elements according to Demographic Characteristics and Usage Behavior Characteristics

A design factor satisfaction analysis was conducted according to gender, age, occupation, and residence. As for the analysis method, two group variables (men and women) were tested for differences through an independent sample *t*-test, and the remaining variables (age, occupation, education, and residence) were compared with the satisfaction of three or more group variables, and an analysis of variance (ANOVA, St. New Providence, NJ, USA) was conducted. ANOVA is a statistical technique used to compare the average values of two or more groups, and the verification statistic at this time is F. Differences in satisfaction with users’ design factors were analyzed under the hypothesis that their thoughts may vary depending on transportation, arrival time, number of uses, seasonal use, day of week use, time of use during the day, stay time, accompanying visits, and purpose. Analysis of variance (ANOVA, St. New Providence, NJ, USA) was performed as the analysis method (X: There is a significant difference, indicating that there is an influence relationship between the two factors. O: There is no significant difference, indicating that there is no influence relationship between the two factors. F: F-value, the F-value is the ratio of the two mean squares (effect term/error term), negative values are not possible).

In Houtan Park, satisfaction with design elements differs by sex, age, transportation, frequency of use, time of use, and reason for visit (Table 14).

Regarding the design element of location characteristics, the respondents in their sixties were the most satisfied, while people in their twenties were the least satisfied. In contrast, taxi users were the most satisfied, and private-car owners were the least satisfied.

Natural and environmental characteristics were rated as the highest satisfaction factor by first-time visitors, whereas people who visited once a month rated these characteristics as the lowest satisfaction factor. Additionally, users who came for a picnic were the least satisfied.

While walking received the highest satisfaction scores for user behavior, the value of using public transportation received the lowest scores. Using the park during the afternoon reflected the highest satisfaction factor, while using it in the evening reflected the lowest. Additionally, those in their thirties were the most satisfied, while those in their sixties were the least satisfied.

Regarding management characteristics, males showed higher satisfaction than females. Users who came to enjoy nature were the most satisfied, and people who came to exercise were the least satisfied.

In Xuhui Binjiang Park, satisfaction with the design elements varies depending on the age, occupation, and reason for visit. Regarding location characteristics, people who were retired and were sixty years old or older exhibited the highest satisfaction, while students in their twenties or younger exhibited the lowest. Additionally, the highest satisfaction levels are observed in the evening, while the lowest satisfaction levels are observed in the morning. Moreover, for the design elements of the location characteristics, the users who came to take pictures showed the highest satisfaction. In contrast, the users who came to participate in events showed the lowest satisfaction (Table 15).

As for usability characteristics, those in their twenties were the most satisfied, while those in their sixties were the least satisfied. People who visited the park in the afternoon showed the highest satisfaction, and those who visited at lunchtime showed the lowest satisfaction.

Regarding administrative characteristics, those in their sixties were the most satisfied, while those in their forties were the least satisfied. Additionally, regarding management characteristics, it was found that the users who came for a walk were satisfied the most and the users who came to enjoy nature showed the lowest satisfaction.

### 4.6. Analysis of the Influence Relationship between Satisfaction and Behavioral Intention

In Houtan Park, natural and environmental characteristics mainly affect the overall satisfaction level, which, in turn, influences the intention to revisit.

The results of multiple regression analysis conducted to analyze the effect of satisfaction with design factors on the overall satisfaction of Houtan Park, revisit intention, and recommendation intention are as follows (Table 16).

As a result of analyzing the effect of satisfaction with design elements on overall satisfaction of Houtan Park, the explanatory power of the regression model was 45.2%, and the regression formula was analyzed to be statistically significant (F = 59.13, *p* < 0.01). The VIF values of all independent variables are less than 10, so it is judged that there is no problem with multicollinearity. For each independent variable, natural environmental characteristics (β = 0.368, *p* < 0.05) were found to have a significant positive (+) effect on overall satisfaction with Houtan Park. In other words, it can be seen that the influence of the natural environmental characteristics on the satisfaction of Houtan Park is large.The results of the analysis of the effect of satisfaction on the pursuit intention of Houtan Park are as follows. It was found to have no statistical effect (*p* > 0.01).

The results of analysis of the effect of satisfaction with design elements of Houtan Park on revisit intention are as follows. It was found to have no statistical effect (*p* > 0.01).

As a result of analyzing the effect of the overall satisfaction of Houtan Park on the revisit intention, the explanatory power of the regression model was 43.1% and the regression equation was analyzed to be statistically significant (F = 53.62, *p* < 0.01). The VIF value of all independent variables is less than 10, so there is no problem with multicollinearity. By independent variable, overall satisfaction (β = 0.213, *p* < 0.05) was found to have a significant positive (+) effect on revisit intention. In other words, it can be seen that there is an influence of overall satisfaction on the intention to revisit Houtan Park

In Xuhui Binjiang Park, the location and usage characteristics affect the overall satisfaction level and the usability characteristics have a significant (+) effect on the intention to revisit. Furthermore, overall satisfaction affected revisit intention.

The results of multiple regression analysis conducted to analyze the effect of satisfaction with design elements of Xuhui Binjiang Park among overall satisfaction, revisit intention, and recommendation intention are as follows (Table 14).

As a result of analyzing the effect of satisfaction with design elements of Xuhui Binjiang Park on overall satisfaction, the explanatory power of the regression model was 52.8% and the regression formula was analyzed to be statistically significant (F = 50.98, *p* < 0.001). The VIF values of all independent variables are less than 10, so it is judged that there is no problem with multicollinearity. By independent variable, location characteristics (β = 0.212, *p* < 0.05) and usability characteristics (β = 0.222, *p* < 0.05) were found to have a significant positive (+) effect on overall satisfaction with Xuhui Binjiang Park. In other words, it can be seen that the influence of location characteristics and usage characteristics has the greatest effect on the satisfaction of Xuhui Binjiang Park.

Table 17 shows the results of analysis on the effect of satisfaction with design elements of Xuhui Binjiang Park on the intention to pursue. It was found to have no statistical effect (*p* > 0.01).

As a result of analyzing the effect of satisfaction with design factors of Xuhui Binjang Park on revisit intention, the explanatory power of the regression model was 73.0% and the regression formula was analyzed to be statistically significant (F = 52.29, *p* < 0.01). The VIF values of all independent variables are less than 10, so it is judged that there is no problem with multicollinearity. By independent variable, utilization characteristics (β = 0.274, *p* < 0.05) were found to have a significant positive (+) effect on the intention to revisit Xuhui Binjang Park. In other words, it can be seen that the usability characteristic has the greatest influence on the intention to revisit Xuhui Binjiang Park.

### 4.7. Problems and Improvement Measures

**Figure 4:**
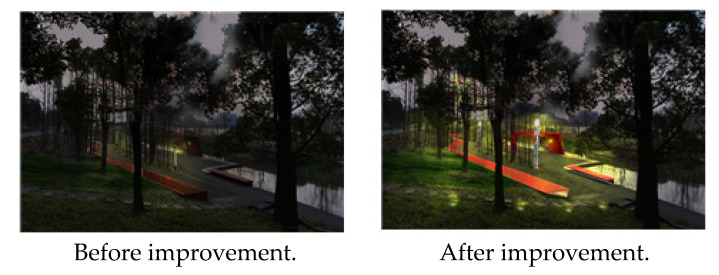
Houtan Park lighting facility. Source: author created.

In the case of Houtan Park, the first major problem to be solved was night safety (security), caused by the aging of the lighting facilities and insufficient safety maintenance. Accordingly, the aging lighting facilities were changed and a focus was placed on continuous safety maintenance and management (Figure 4).

Second, user satisfaction is low due to inadequate transportation access. Institutional arrangements—such as more bus routes that arrive near Houtan Park or rentable bicycles from subway stations to parks—are needed.

**Figure 5:**
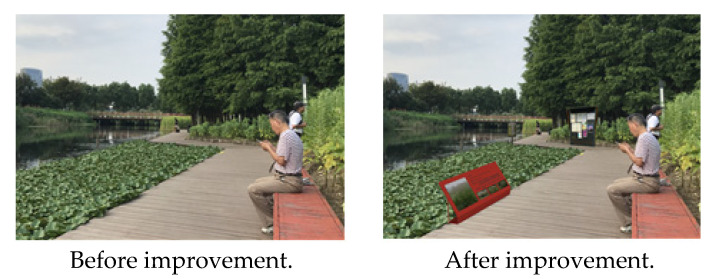
Houtan Park information facility. Source: author created.

Third, the information facilities are insufficient and must be expanded; the visitor center, which has a guiding function, is used by few people. The rarely installed used bulletin boards do not adequately convey information about the facilities and habitats in Houtan Park (Figure 5).

**Figure 6:**
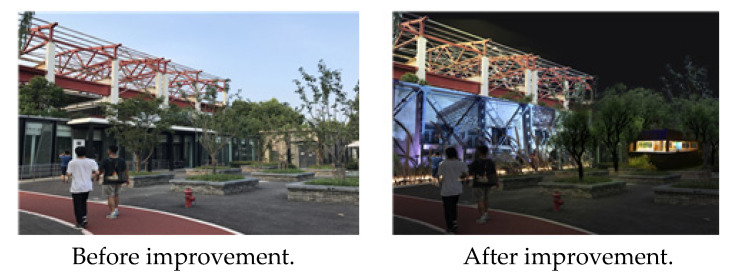
Houtan Park industrial heritage program. Source: author created.

Fourth, the lack of convenience facilities in the park can be expanded to increase users’ satisfaction. Fifth, various programs should be prepared because the current program operated at Houtan Park exhibited the lowest satisfaction level. Sixth, the importance of the park’s industrial heritage culture can be recognized by actively promoting the symbolic facilities and buildings preserved in Houtan Park, using information facilities or programs (Figure 6).

**Figure 7:**
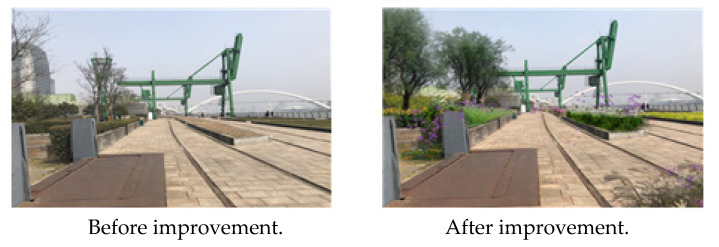
Xuhui Binjiang Park plants. Source: author created.

In Xuhui Binjiang Park, first, the water quality management of the dock garden was problematic; thus, continuing to monitor water quality is necessary. Second, the visitor center, which is in charge of guidance functions, has a low usage rate. There are information boards that are rarely used and do not adequately convey information about the facilities in Xuhui Binjiang Park; hence, sufficient information facilities must be introduced. Third, since the types of plants are unified, a greater variety of trees and flowers are needed. In particular, the planting and water-friendly areas growing at the time of the ruins require more aquatic plants than they have now (Figure 7).

**Figure 8:**
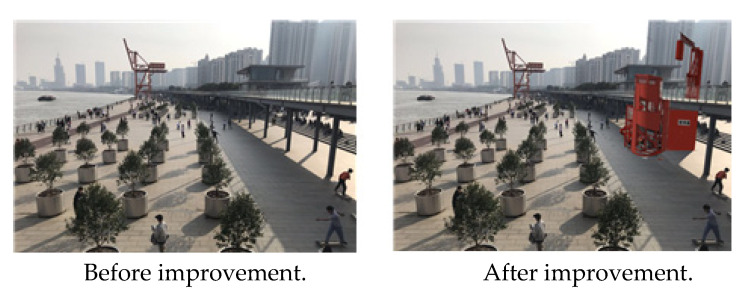
Xuhui Binjiang Park plants. Source: author created.

Fourth, the introduction of convenience stores and toilets, which are currently insufficient in the park, will increase users’ intentions to visit and increase their satisfaction level (Figure 8).

Fifth, strengthening the management of underdeveloped facilities and plants is necessary to improve park maintenance. Sixth, various programs should be prepared because the usability satisfaction was the lowest due to the lack of programs currently operated by Xuhui Binjiang Park. In particular, the active promotion of local history and industrial heritage culture is needed through programs and activities.

## 5. Discussion

With the rapid development of urban construction, the waterfront industrial heritage park has played an active role in shaping the city’s image, regional economic development and environmental improvement, and the continuation of the city’s waterfront history. The waterfront park based on industrial heritage using the post-occupancy evaluation will help to improve the sustainable management, maintenance, and design level of the project in the future. However, there is insufficient research on the waterfront industrial heritage park using the post-occupancy evaluation. Looking back on the research on the transformation of China’s industrial heritage into parks so far, it is almost only on the basis of theoretical research, and then through the case analysis of domestic and foreign industrial heritage parks, the problem with transforming China’s industrial heritage is drawn. However, most studies are only focused on the design side, and studies on the statistical analysis side are insufficient. Looking at previous studies on the post-use evaluation of Chinese industrial heritage-based parks, it is mainly limited to the frequency analysis of users’ usage patterns and satisfaction with facilities, regardless of the post-use evaluation studies of general parks.

This paper takes Shanghai Houtan Park and Xuhui Binjiang Park, the representative industrial heritage parks in China, as the research objects. Through field investigation and nearly 200 questionnaires and interviews regarding user behavior, the importance of design elements (place characteristics, natural environmental characteristics, usability characteristics, and administrative characteristics ) and the correlation of satisfaction helps us understand the use of the two parks and analyze and organize the survey data, carrying out the analysis of questionnaire results using frequency analysis, IPA analysis, t-test, variance analysis, and multiple regression analysis.

## 6. Conclusions

This study suggests problems and supplementary measures by comparatively analyzing the post-occupancy evaluations of Houtan Park and Xuhui Binjiang Park in Shanghai, which are representative examples of China’s industrial heritage-based waterfront parks. It also aims to present primary data that should be considered in future waterfront parks based on industrial heritage. The conclusions of this study are summarized as follows.

First, variables to be considered as factors for post-use evaluation of industrial heritage-based waterfront parks are mainly divided into four types: site characteristics, natural environmental characteristics, usage characteristics, and administrative characteristics.

Second, in both parks, the prominent users were in their twenties and forties, and the walking trail showed the highest use rate as the main facilities. In contrast, the visitor centers, which are in charge of the guidance function, showed the lowest usage rate.

Third, the descriptive statistical analysis showed that although the two parks commonly focus on the natural and environmental characteristics and management characteristics for the importance of design elements, site characteristics are not of central importance. In terms of satisfaction with the design elements of Houtan Park, natural environmental characteristics exhibited high satisfaction. However, the site characteristics, usage characteristics, and administrative characteristics received low evaluations. Although the site and utilization characteristics of Xuhui Binjiang Park were found to be relatively satisfactory, users were not satisfied with the natural and environmental characteristics and administrative characteristics.

Fourth, currently, Houtan Park, viewed through IPA analysis, exhibited the following most urgent problems to be solved: improving accessibility, programs, information facilities, amenities, lighting facilities, and safety management from management characteristics. For Xuhui Binjiang Park, the improvement of local history reflection, plant diversity, polluted water purification, programs, information facilities, convenience facilities, facilities maintenance, and plant ecology maintenance were the most urgent problems to resolve.

Fifth, through the analysis of design element satisfaction according to user characteristics and usage behavior characteristics, in Houtan Park, differences were found in design element satisfaction—mainly according to sex, age, transportation method, frequency of use, time of use, and reason for visit. In Xuhui Binjiang Park, satisfaction with design elements mainly differed according to age, occupation, the time of day, and the reason for visit.

Sixth, the analysis of the impact relationship between design element satisfaction and behavior showed that the natural and environmental characteristics of Houtan Park affect overall satisfaction and revisit intention. In Xuhui Binjiang Park, the site and utilization characteristics affect the overall satisfaction and the utilization characteristics have a positive effect on the intention to revisit. Furthermore, there was a high intention to revisit.

The following inspirations are drawn from the conclusions.

First, when designing waterfront parks based on Chinese industrial heritage, it is necessary to realize not only that the complex value of regional identity must be secured through industrial heritage, but also that the artificial landscape and ecological environment can be organically combined. Give full attention to the advantages of green open space as a waterfront living carrier. Planning and construction cannot simply meet the needs of greening visual landscapes. It should be fully integrated into the city’s green infrastructure system, integrated with people’s ecological demands, transportation, sports and leisure activities, and create a new carrier for waterfront life. The green open space forms a good connection between the city and the water area, improves the regional ecological environment, and meets people’s needs for outdoor leisure and place perception, so that people of different classes in the city can live in harmony and feel the charm of waterfront life together. New York’s Hudson River Park is a representative example of the renewal and development of waterfront industrial zones led by the open space model. Hudson River Park is an important part of New York City’s park system and the core area of New York’s greenway system. As the green infrastructure of the city, the park passes through many blocks and landmark buildings and connects the urban open space on the west side of Manhattan Island into a green whole. The north–south bicycle path on the west side of the elevated highway completes the transition and connection with the urban interface, and together with the waterfront pedestrian path, it has become a necessary carrier for urban slow-moving life. There are various themed leisure venues and recreational sports facilities in the park, such as sunny lawns, water piers, outdoor courts, small restaurants, and pet parks, etc., providing green spaces and places for individual and collective activities, life leisure, and children’s play [41]. In order to evoke people’s unique memory of the place, the landscape facilities meet the diverse needs of waterfront leisure.

Second, use the contrast between waste and fashion to realize the organic combination of site shaping and site emotion, so that everyone has the possibility to resonate with the scene. The city’s waterfront industrial zone has witnessed the civilization of human beings in the industrial period and also carries the unique memory of production and life. Therefore, its renewal and development cannot be just a simple new construction or reconstruction. A scientific assessment of valuable landscape resources should be carried out and a mechanism for coexistence of protection and development should be formed, so that the creation of open space can not only meet the needs of green life, but also arouse people’s exclusive emotions about the place. Simple industrial relics will not become a tourist hotspot. The key is to achieve deep and high-quality integration of industrial heritage with culture, art, entertainment, etc., and the diversification of operation modes can make people willing to visit here. As the venue for the skiing competition of the 2022 Winter Olympics, Beijing Shougang Park has become a composite organic space, integrating art, architecture, urban history, and technological innovation. Introduce skateboarding, rock climbing, and other activities, create a training brand base and trendy event IP, and use the industrial heritage of the site to create a virtual reality museum, immersive theater, VR e-sports hall, Olympic project experience center, future light and shadow interactive restaurant, and other consumption activities. The business format also attracts a large number of young people. Using scientific and technological means, through modern media art means, can help restore the production and living scenes and encourage tourists to interact with the current scenic spots [13].

Third, pay attention to the development of industrial heritage-themed tour paths. When designing an industrial heritage-based waterfront park, strengthening transportation accessibility or traffic connections in the surrounding area is necessary. For example, the world-famous German “Industrial Heritage Tourism Road” will actually transform the industrial heritage of the Ruhr industrial area in Germany and connect it with traffic nodes to introduce tourists to Germany’s coal and steel history that spans hundreds of years. The evolution of industrial civilization, such as a textbook of industrial civilization, is no longer a point of tourism, but a tourist route [42]. It is also possible to consider the transportation mode of cruise ships, connecting Houtan Park and Xuhui Binjiang Park to form a tourist route.

Fourth, industrial heritage-based waterfront parks are dynamic and constantly evolving with urban and social development. Therefore, park maintenance is essential; hence, establishing a professional management department—comprising public–private organic cooperation, residents, experts, artists, and historians—is necessary to review the park’s maintenance plan. In particular, continuous media communication with users—for example, through the park’s web page—is important.

Fifth, establish and improve relevant laws and special plans for the renewal and development of waterfront industrial heritage. China’s current national and local laws and regulations still lack special provisions for the protection and development of urban industrial zones or industrial heritage, and the planning of waterfront areas is also relatively lagging and one-sided due to various factors. This restricts the effective development of a waterfront industrial zone. New York’s waterfront space revival plan and a series of bills such as the Hudson River Park Act provide a good reference. Relevant laws and regulations should be established and improved so that the renewal and development work can be legally based, and scientific and reasonable formulations should be formulated according to the actual needs of different regions. In addition, special planning to provide upper-level basis and theoretical support is needed [43].

## Figures and Tables

**Table 1:** Cases of waterfront industrial heritage parks.

Case	Parc de La Villette ¹	Hudson River Park ²	IBA Emscher Park ³	Thames Barrier Park ⁴	Sunyudo Park ⁵	Shougang Park ⁶
Project location	Paris, France	New York, United States	Ruhr, Germany	London, United Kingdom	Seoul, South Korea	Beijing, China
Waterfront	Canal de Saint-Denis	Hudson River	Ruhr River, Lippe River	Thames River	Han River	Yongding River
Construction time	1982–1988	1986–present	1989–2010	1995–2000	2001–2002	2018–2022
Scale	0.55 km^2^	2.2 km^2^	800 km^2^	0.09 km^2^	0.11 km^2^	0.7 km^2^
Original function	Slaughterhouse	Pier	Coal plant, steelworks	Chemical plant, pier	Water treatment plant	Steelworks
Design strategy	A city where science and music can meet, a garden in the city [8].	“Edge, channel, movement and island” to evoke the unique memory of the place, and the landscape facilities meet the diverse needs of waterfront leisure [9].	A new urban waterfront that incorporates different activities and brings back the water within the city [10].	A park that is clearly woven into its surroundings [11].	Landscape must be ecological, functional, and beautiful, and the traces of industrial history cannot be erased [12].	Create a micro-vacation experience [13].
Specific measure	The overlapping of the three elements of point, line, and surface in the park forms a spatial system that connects the city and the park [14].	Hudson River Park was established in 1998 through the Hudson River Park Act, pier transformations, plan to create an 8km green open space [9].	The Industrial Heritage Trail: Designed a system of bike paths, parks, and green belts across 17 cities for extensive regional connectivity [15]	The emphasis was on structurally and visually connecting the development area north of the park site, the Thames River in the south, and flood prevention facilities [11].	Retain the original industrial heritage space to show the water purification cycle [16].	Change its use function on the premise of retaining the original structure and external appearance of the industrial heritage to the greatest extent [17].
Functions and facilities	Points are 10 m high red structures arranged at grid intersections 120 m apart in the park, and are used for various purposes such as cafes, emergency centers, and information centers. Lines are an orthogonal system for high-density pedestrian traffic that crosses the entire park in a cross. Surfaces provide an open space with a wide horizontal surface to accommodate all activities of visitors, including grass squares, sports grounds, and theme gardens [18].	Industrial heritage sites and facilities are planned for various uses such as public recreation as well as maritime, municipal, and commercial. There are various themed leisure venues and recreational sports facilities in the park, such as sunny lawns, water piers, outdoor courts, small restaurants, and pet parks, etc., providing green spaces and places for individual and collective activities, life leisure, and children’s play [19].	Transform the brownfields in an infrastructure of open spaces and creative activities: the use of industrial heritage to create new natural areas, integration of industrial heritage into parks, and transformation of industrial heritage into functional buildings (exhibition halls, museums, educational centers, churches, creation of commercial, recreational facilities, office, or residential areas) [10].	The park offers 32 fountains, children’s playgrounds, and picnic areas. In addition, the 130-foot-long “Green Dock” diagonally across the park provides wind-protected microclimate for a variety of plants and wildlife [11].	Various facilities such as Han River Exhibition Hall, Tourist Information Center, Greenhouse, Environmental Classroom, Green Column Garden, Aquatic Botanical Garden, Small Theater, etc., are designed as a place for ecological environment education and experience by retaining industrial facilities [20].	Transform the brownfields in an infrastructure of open spaces and creative activities: the use of industrial heritage to create new natural areas, integration of industrial heritage into parks, and transformation of industrial heritage into functional buildings (extreme sports venues, national team training venues, museums, shopping malls, restaurants, cafes, hotels, modern office spaces) [13].
Activities	Education workshops, exhibitions, fairs, workshops, and park tours are free of charge [14].	Cultural and fitness events, active and passive recreation, educational and environmental programs, and Circle Line Sightseeing Cruises [21].	Through the use of marketing and the media (light installations events, music festivals, art shows) and an intense work of branding [22].	Children can run and play happily here; the elderly can walk slowly and leisurely. It provides a relaxed, quiet, and ecological free space for all people [23].	Learn the natural ecology and history of the Han River, conferences, exhibitions, performances, etc. [24].	Organizing events (such as the 2022 Beijing Winter Olympics, extreme sports competitions, clothing exhibitions, etc.) [17].

¹ [8,14,18]; ² [9,19,21]; ³ [10,15,22]; ⁴ [11,23]; ⁵ [12,16,20,24]; ⁶ [13,17].

**Table 2:** Design elements of waterfront parks based on industrial heritage.

Item	Design Elements	Hu Yan ¹	He Wang ²	Wang Xiao Jia ³	Yang Zhen yu ⁴	Liu Wen Li ⁵	Liu Qin ⁶	Zhu Yi Chen ⁷	Li Shuang ⁸	Hong Qing Qian ⁹	Huang Kun Yin ¹⁰	Wang Min ¹¹	Wu Dan Zi ¹²	Total
Place characteristics	Fragmental preservation of existing buildings	◉					◉		◉					3
	Abandoned facilities regenerated as parks	◉		◉	◉		◉	◉		◉		◉	◉	8
	Utilized as the main building in the entire park design	◉		◉	◉			◉		◉				5
	An object that symbolizes an existing place		◉					◉		◉				3
	Recycling waste	◉		◉		◉		◉				◉		5
	Preservation of symbolic structures	◉		◉	◉	◉		◉		◉	◉	◉		8
	Preservation and re-creation of landmarks	◉			◉		◉	◉	◉	◉				6
	Preservation of existing environmental characteristics		◉		◉						◉			3
	Conservation of symbolic facilities	◉	◉	◉	◉	◉	◉		◉		◉	◉	◉	10
	Restoration time before the relocation of the facility	◉								◉				2
	Reproduction of local traditional elements		◉				◉		◉					3
	Reflecting local history through activities	◉			◉		◉	◉	◉		◉	◉		7
Natural environmental characteristics	Preserved wild plants	◉			◉		◉	◉						4
	Introduced hydrostatic plants and native plants					◉			◉					2
	Regeneration of damaged/neglected areas	◉			◉		◉		◉	◉	◉	◉		7
	Connecting the surrounding greenery		◉		◉		◉	◉	◉	◉	◉	◉		8
	Flood prevention								◉		◉			2
	Wetlands					◉		◉		◉				3
	Green area	◉			◉	◉		◉						4
	Contaminated soil purification	◉	◉		◉		◉			◉			◉	6
	Polluted water purification	◉		◉		◉				◉		◉	◉	7
Usability characteristics	Information facilities	◉	◉		◉		◉		◉		◉	◉	◉	8
	Resting facilities			◉		◉				◉				3
	Amenities		◉		◉		◉		◉	◉				5
	Program		◉	◉		◉		◉		◉	◉	◉		7
	Landscape facilities			◉	◉		◉		◉			◉	◉	6
	Cultural facilities	◉	◉		◉		◉		◉	◉	◉			7
	Sports facilities		◉	◉				◉		◉	◉	◉		6
	Lighting facilities	◉				◉		◉	◉		◉		◉	6
	Accessibility		◉	◉		◉		◉		◉	◉		◉	7
	Activity area	◉		◉				◉		◉	◉			5
	Site path			◉				◉		◉	◉	◉		5
Administrative characteristics	Facility maintenance	◉				◉			◉		◉		◉	5
	Vegetation maintenance	◉			◉				◉	◉		◉	◉	6
	Industrial heritage maintenance		◉		◉	◉	◉		◉		◉	◉		7
	Clean facilities	◉			◉				◉	◉		◉	◉	6
	Secure	◉			◉				◉			◉	◉	5

¹ [28]; ² [29]; ³ [30]; ⁴ [31]; ⁵ [32]; ⁶ [33]; ⁷ [34]; ⁸ [35]; ⁹ [36]; ¹⁰ [37]; ¹¹ [38]; ¹² [39].

**Table 3:** Evaluation index of waterfront parks based on industrial heritage.

Characteristic	Design Elements	Characteristic	Design Elements
Place characteristics	Recycling waste facilities into park facilities	Usability Characteristics	Information facilities
	Recycling waste into park design elements		Activity space
	Preservation and re-creation of surface traces		Cultural facilities
	Preservation of symbolic facilities and buildings		Amenities
	Reflect local history through activities		Program
			Sports facilities
			Landscape facilities
			Lighting facilities
			Accessibility
			Site path
Natural environmental characteristics	Connectivity of surroundings	Administrative characteristics	Facility maintenance
	Harmonious natural scenery		Landscape maintenance
	Purification of contaminated water		Industrial heritage maintenance
	Plant diversity		Security
	Regeneration of the environment in neglected or damaged areas		Cleanliness

**Table 4:** Overview of the study site.

Park Name	Houtan Park	Xuhui Binjiang Park
Location	2200 Expo Avenue, Pudong New Area, Shanghai	99 Luining avenue, Xuhui District, Shanghai
Area	182,000 m^2^	190,000 m^2^
Construction year	2007	2009
Previous	Huangpu River coastal industrial zone (wharf, warehouse, ship repair plant, steel mill, etc.)	Huangpu River coastal industrial zone (wharf, warehouse, cement mill, paper mill, etc.)
Founding background	“Shanghai 2010 Expo Green Space” Plan Project	“Shanghai 2010 Expo Green Space” Plan Project
Status details	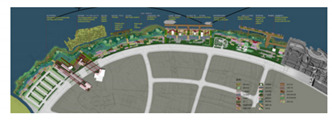 Houtan Park mast plan. Source: https://www.turenscape.com/en/project/detail/443.html (accessed on 16 May 2021). Houtan Park, built on the site of a comprehensive industrial heritage site such as a dock, warehouse, ship repair factory, and steel factory in the past, is located along the east bank of the Huangpu River in Shanghai. Currently, around the park, there is a financial company, a gymnasium, and the buildings of the national pavilion left after the Expo. After introducing regeneration measures such as artificial wetlands, ecological flood control, recycling of abandoned industrial structures and materials, and urban agriculture, the park is purifying the polluted river and restoring the devastated waterfront A linear artificial wetland with a length of 1.7 km and a width of 5 to 30 m passing through the center of the park was designed to become a waterside space that purifies the polluted water flowing in from the Huangpu River with a living device The cascades and terraces oxygenate the eutrophic waters to remove nutrients and remove sediment while making the park a revitalizing hydroponic facility. In addition, various types of wetland plants were planted here to absorb various types of pollutants [39]. In total, 2400 tons of water per day has been lowered from Level 5 to Level 3. It can be used safely as water, and compared to the existing water purification method, it saves USD 500,000 in cost, and the wetland in the park functions as a buffer to prevent flooding every 20 to 1000 years [40]. The valley curved along the wetland is visually interesting and becomes a resting place for leisure activities such as walking and recreation. The terrace design of the wetland alleviates the elevation difference between the city and the river and safely connects people and the waterfront. In addition, the existing concrete embankment was replaced with a crushed stone shoreline closer to the habitat to protect the riverbank from erosion and to encourage native species to grow along the riverbank. Currently, Houtan Park is managed by Shanghai Expo Cultural Tourism Development Co., Ltd	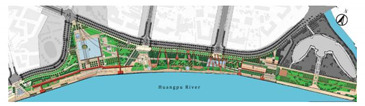 Xuhui Binjiang Park mast plan. Source: https://www.sohu.com/a/158235536_155927 (accessed on 16 May 2021). Xuhui Binjiang Park, which was built on a comprehensive industrial heritage site such as a pier, warehouse, cement factory, and paper factory in the past, is located along the west bank of Shanghai’s Huangpu River. There are mainly residential areas and financial companies around the park. The park’s design goal is to reinterpret the place’s industrial heritage and cultural memories to create a natural past, present, and future connection between the city, people, and waterfront. By preserving and recycling the symbolic facilities and structures left on the site, the theme of the park was emphasized and its activity was increased. The freight transport railroad left at the pier is preserved and it is designed as an activity line linking the entire park, and materials such as mooring poles and railroad ties are recycled as chairs in the park. As a landscape sculpture with the structure of a 36m crane maintained as it is, the night view is formed through night LED projection, and the dock unloading facility is converted into an art museum and various art exhibitions are held every year. The factory building at the pier has been converted into a planning and exhibition center. In addition, the cargo transmission facility from the cargo ship on the river to the land was left intact, and it was converted into a 420m public walking path that offers a view of the Huangpu River. Under the structure of the public walkway is a space where visitors can experience climbing exercise outdoors as well as providing shade and a rest area to take cover from the rain. In addition, facilities capable of mist spraying can be installed to lower the temperature during a heat wave [29]. The inside of the park is designed as a skateboard space and water garden in the form of a sunken garden with a difference of 1.8m in depth. Rainwater is collected and recycled through the pitched road surface and rainwater garden, and a windmill using wind power is installed on the slope. It is used to supply power for lighting in the park. Currently, Xuhui Binjiang Park is managed by Shanghai Xi’an Development Group Co., Ltd. Every year, Xuhui Binjiang Park hosts activities such as a funeral, an idea market, and a marathon. Citizens also voluntarily engage in various club activities such as fitness, dancing, running, rock climbing, and skateboarding in this park [39].

**Table 5:** Post-occupancy evaluation questionnaire regarding the composition of industrial heritage-based parks.

Item	Evaluation Items	Main Content	Number of Questions	Evaluation Contents	Evaluation Scale
Design elements of waterside park using industrial heritage	Location characteristics	Recycling waste facilities into park facilities, recycling waste into park design elements, preserving and re-creating surface traces, preserving iconic facilities and structures, reflecting local history through activities	5	Importance and satisfaction	Five-point Likert scale
	Natural environmental characteristics	Connectivity of the surrounding environment, pleasant natural scenery, purification of polluted water quality, plant diversity, environmental regeneration of neglected and damaged areas	5		
	Usability characteristics	Information facilities, activity space, cultural facilities, program, amenities, sports facilities, landscape facilities, lighting facilities, accessibility, site path	10		
	Administrative characteristics	Facility maintenance, tree maintenance, industrial heritage maintenance, safe maintenance, clean maintenance	5		
Action intention		Overall satisfaction, revisit intention, recommendation intention	3		
User behavior analysis	User behavior analysis	Means of transportation, arrival time, frequency of use, seasonal use, day of week of use, time of use, stay time, visit companions, reason for visit, mainly used place, most used facility	11	-	Nominal
Demographic characteristics	User general status	Age, sex, occupation, education, place of residence	5	-	Nominal
What must be improved	Open-ended questions				

**Table 6:** Reliability analysis of the preliminary survey.

Content	Item	Alpha Value	Number of Questions
**Waterfront park based on industrial heritage**	Importance	0.925	25
	Performance	0.819	25
	Action intention	0.823	3

**Table 11:** Reliability of design element importance and satisfaction.

Item	Houtan Park Cronbach’s Alpha Value		Xuhui Binjiang Park Cronbach’s Alpha Value	
	Importance	Satisfaction	Importance	Satisfaction
Preservation and re-creation of surface traces	0.818	0.808	0.843	0.827
Preservation of symbolic facilities and buildings	0.811	0.814	0.843	0.835
Recycling waste facilities into park facilities	0.804	0.806	0.844	0.828
Recycling waste into park design elements	0.819	0.804	0.844	0.848
Reflect local history through activities	0.824	0.813	0.844	0.822
Harmonious natural scenery	0.820	0.823	0.842	0.829
Regeneration of the environment in areas damaged or neglected	0.801	0.828	0.844	0.838
Plant diversity	0.804	0.801	0.843	0.841
Purification of contaminated water	0.822	0.807	0.845	0.830
Connectivity of surroundings	0.819	0.807	0.842	0.834
Easy to access	0.821	0.803	0.845	0.820
Activity areas	0.822	0.816	0.844	0.824
Circulation	0.814	0.810	0.844	0.836
Program	0.802	0.800	0.844	0.829
Information facilities	0.818	0.802	0.844	0.831
Amenities	0.813	0.807	0.844	0.831
Cultural facilities	0.880	0.804	0.843	0.822
Sports facilities	0.817	0.801	0.846	0.829
Landscape	0.845	0.826	0.852	0.819
Lighting	0.815	0.808	0.843	0.823
Cleanness	0.824	0.819	0.853	0.823
Security	0.817	0.802	0.853	0.825
Facility maintenance	0.819	0.809	0.851	0.821
Plant maintenance	0.815	0.816	0.851	0.834
Industrial heritage maintenance	0.824	0.805	0.855	0.820
All previous	0.819	0.809	0.851	0.828

**Table 12:** Design element importance and satisfaction descriptive statistics.

Division		Houtan Park (Average)				Xuhui Binjiang Park (Average)			
Variable		Importance	Rank	Satisfaction	Rank	Importance	Rank	Satisfaction	Rank
Site	Preservation and re-creation of surface traces	3.28	25	3.05	20	3.65	25	3.45	12
	Preservation of symbolic facilities and buildings	3.78	19	3.54	8	4.03	20	3.78	5
	Recycling waste facilities into park facilities	3.73	21	3.22	13	3.91	22	3.47	11
	Recycling waste into park design elements	3.52	24	3.09	17	3.74	24	3.23	15
	Reflect local history through activities	3.87	17	3.08	18	4.2	13	3.53	10
Natural environmental characteristics	Harmonious natural scenery	4.31	3	4.24	1	4.82	1	3.68	6
	Regeneration of the environment in damaged or neglected areas	4.39	1	4.21	2	4.3	9	3.34	13
	Plant diversity	4.25	7	3.88	6	4.28	10	3.09	19
	Purification of contaminated water	4.24	8	3.98	4	4.33	6	2.98	22
	Connectivity of surroundings	4.04	12	4.00	3	4.14	16	3.61	9
Usability characteristics	Easy to access	3.95	16	3.06	19	4.11	17	3.22	16
	Activity areas	3.77	20	3.33	11	4.16	15	3.83	3
	Easy to navigate the park	4.04	12	3.95	5	4.23	12	3.82	4
	Program	3.96	15	2.79	25	4.27	11	2.83	25
	Information facilities	4.05	10	2.82	24	4.33	6	2.83	25
	Amenities	4.07	9	3.11	15	4.33	6	3.03	20
	Cultural facilities	3.99	14	2.84	22	4.11	17	2.87	23
	Sports facilities	3.57	23	3.16	14	4.05	19	3.64	7
	Landscape	3.66	22	3.11	15	3.81	23	3.27	14
	Lighting	4.05	11	2.97	21	4.2	13	3.92	2
Administrative characteristics	Cleanliness	4.27	6	3.66	7	4.36	5	3.99	1
	Security	4.29	5	3.25	12	4.38	4	3.64	7
	Facility maintenance	4.35	2	3.38	10	4.53	2	3.14	18
	Plant maintenance	4.30	4	3.45	9	4.53	2	3.18	17
	Industrial heritage maintenance	3.86	18	2.84	22	3.96	21	3.00	21

**Table 13:** The numbers from 1 to 25 in the Figure 2 and Figure 3 correspond to the project.

1. Preservation and re-creation of surface traces		2. Preservation of symbolic facilities and buildings	
3. Recycling waste facilities into park facilities		4. Recycling waste into park design elements	
5. Reflect local history through activities		6. Harmonious natural scenery	
7. Regeneration of the environment in areas damaged or neglected		8. Plant diversity	
9. Purification of contaminated water		10. Connectivity of surroundings	
11. Easy to access	12. Activity areas	13. Circulation	14. Program
15. Information facilities	16. Amenities	17. Cultural facilities	18. Sports facilities
19. Landscape	20. Lighting	21. Cleanness	22. Security
23. Facility maintenance	24. Plant maintenance	25. Industrial heritage maintenance	

**Table 14:** Analysis of differences in satisfaction with design elements according to demographic characteristics and usage behavior characteristics in Houtan Park.

Differences in satisfaction according to the demographic characteristics (Houtan Park)	Item	Mean square	Significance probability	Satisfaction difference								
	Sex	0.210	0.011	O								
	Age	0.120	0.036	O								
	Job	0.041	0.222	×								
	Educational level	0.036	0.325	×								
	Residence	0.030	0.446	×								
Differences in satisfaction by sex (Houtan Park)	Variable	Male (average)	Female (average)	*t*-value	Significance probability							
	Administrative attributes	3.51	3.12	2.496	0.023							
Differences in satisfaction by age (Houtan Park)	Variable	Under 20	20s	30s	40s	50s	Over 60	F	Significant probability			
	Site characteristics	2.91	3.11	3.13	3.27	3.33	3.56	4.280	0.000			
	Usable characteristics	3.34	3.36	3.53	3.28	3.23	3.08	3.753	0.018			
Differences in satisfaction by usage (Houtan Park)	Item	Mean square	Significance probability	Satisfaction difference								
	Transportation	0.594	0.022	O								
	Arrival time	0.952	0.438	×								
	Frequency of Use	0.870	0.033	O								
	Seasonal use	0.040	0.670	×								
	Use by day	0.103	0.816	×								
	Use by time	0.030	0.030	O								
	Stay time	0.654	0.582	×								
	Accompanying visit	0.075	0.058	×								
	Reason for visit	0.034	0.015	O								
Differences in satisfaction by transportation methods in Houtan Park	Variable	By foot	Public transport	Private car	Bicycle	Taxi	Others	F	Satisfaction difference			
	Site characteristics	3.20	3.12	3.00	3.06	3.60	-	3.513	0.010			
	Usable characteristics	3.46	3.02	3.07	3.23	3.11	-	2.631	0.038			
Differences in satisfaction by frequency of use (Houtan Park)	Variable	Everyday	Once every two–three days	Once a week	Once a month	Once per season	Once in 6 months	Once a year	First visit	F	Satisfaction Difference	
	Natural environmental characteristics	4.22	3.88	4.06	3.56	4.17	4.20	4.05	4.25	2.37	0.03	
Differences in satisfaction by time of use (Houtan Park)	Variable	Morning	Lunchtime	Afternoon	Night	Regardless of the time	F	Satisfaction difference				
	Usable characteristics	3.21	3.10	3.43	2.99	3.03	4.033	0.005				
Differences in satisfaction by reason for visit (Houtan Park)	Variable	Walk	Exercise	Rest	Picnic	Photography	Attending events	Enjoy nature	Dating	Learning about natural ecology	F	Satisfaction difference
	Natural environmental characteristics	3.98	3.92	4.20	3.71	4.13	3.93	4.33	4.00	4.42	2.16	0.05
	Administrative characteristics	3.44	3.03	3.51	3.22	3.31	3.12	3.67	3.23	3.53	2.03	0.02

**Table 15:** Analysis of differences in satisfaction with design elements according to demographic characteristics and usage behavior characteristics in Xuhui Binjiang Park.

Differences in satisfaction according to the demographic characteristics (Xuhui Binjiang Park)	Item	Mean square	Significance probability	Satisfaction difference								
	Sex	1.021	0.081	×								
	Age	0.083	0.021	O								
	Job	0.081	0.006	O								
	Educational level	0.132	0.000	×								
	Residence	0.310	0.450	×								
Differences in satisfaction by age (Xuhui Binjiang Park)	Variable	Under 20	20s	30s	40s	50s	Over 60	F	Satisfaction difference			
	Site characteristics	3.24	3.26	3.35	3.38	3.51	3.86	2.655	0.033			
	Usable characteristics	3.67	3.72	3.61	3.22	3.23	3.04	2.888	0.018			
Differences in satisfaction by occupation (Xuhui Binjiang Park)	Variable	Student	Public employee	Private employee	Homemaker	Self-employment	Technician	Professor	Retire	Others	F	Satisfaction difference
	Site characteristics	3.02	3.55	3.75	3.37	3.48	3.63	3.65	3.73	3.23	2.356	0.043
	Usable characteristics	3.45	3.32	3.46	3.10	3.25	3.22	3.42	4.00	3.13	2.817	0.022
Differences in satisfaction by usage (Xuhui Binjiang Park)	Item	Mean square	Significance probability	Satisfaction difference								
	Transportation	0.049	0.192	×								
	Arrival time	0.033	0.869	×								
	Frequency of use	0.035	0.362	×								
	Seasonal use	0.012	0.781	×								
	Use by day	0.059	0.114	×								
	Use by time	0.015	0.017	O								
	Stay time	0.018	0.647	×								
	Visit companion	0.031	0.425	×								
	Reason for visit	0.300	0.048	O								
Differences in satisfaction by time of use (Xuhui Binjiang Park)	Variable	Morning	Lunchtime	Afternoon	Night	Regardless of time	F	Satisfaction difference				
	Site characteristics	3.13	3.39	3.55	3.86	3.53	3.718	0.000				
	Usable characteristics	3.13	3.02	3.63	3.42	3.43	3.083	0.023				
Differences in satisfaction by reason for visit (Xuhui Binjiang Park)	Variable	Walk	Exercise	Rest	Picnic	Photography	Attending and event	Enjoy nature	Natural ecology learning	F	Satisfaction difference	
	Site characteristics	3.50	3.72	3.42	3.53	3.96	3.11	3.56	3.33	2.078	0.026	
	Usable characteristics	3.56	3.49	3.28	3.32	3.48	3.24	3.02	3.21	4.522	0.012	

**Table 16:** Analysis of the relationship between satisfaction and behavioral intention of Houtan Park.

	Independent Variables		Non-Standard Coefficient		Standard Coefficients	*t*	Satisfaction Difference	Collinearity Statistics	
			B	Standard Error	Beta			Tolerance	VIF
Relationship between satisfaction of design elements and overall satisfaction of Houtan Park (R²: 0.452, F-value: 59.13, *p*-value: 0.000)	Design elements	(Constant)	1.375	0.719		1.911	0.059		
		Site properties	−0.011	0.155	−0.007	−0.07	0.944	0.89	1.124
		Natural environmental characteristics	0.346	0.091	0.368	3.818	0.000	0.93	1.075
		Usage characteristics	0.35	0.133	0.255	2.637	0.15	0.928	1.078
		Administrative characteristics	0.08	0.088	0.087	0.905	0.368	0.947	1.056
Relationship between satisfaction of design elements and recommendation intention of Houtan Park (R²: 0.245, F-value: 19.13, *p*-value: 0.0.218)	Design elements	(Constant)	5.362	1.539		3.485	0.001		
		Site properties	−0.064	0.359	−0.021	−0.178	0.859	0.766	1.306
		Natural environmental characteristics	0.095	0.182	0.054	0.518	0.606	0.939	1.064
		Usage characteristics	−0.077	0.351	−0.025	−0.219	0.827	0.781	1.28
		Administrative characteristics	−0.396	0.206	−0.199	−1.925	0.057	0.966	1.035
Relationship between satisfaction of design elements and revisit intention of Houtan Park (R²: 0.280, F-value: 0.530, *p*-value: 0.622)	Design elements	(Constant)	3.005	1.525		1.97	0.052		
		Site properties	0.108	0.356	0.036	0.304	0.762	0.766	1.306
		Natural environmental characteristics	−0.026	0.181	−0.015	−0.143	0.886	0.939	1.064
		Usage characteristics	0.372	0.348	0.125	1.07	0.288	0.781	1.28
		Administrative characteristics	−0.066	0.204	−0.034	−0.324	0.747	0.966	1.035
Relationship between the overall satisfaction and Revisit intention of Houtan Park (R²: 0.431, F-value: 53.62, *p*-value: 0.000)	-	Constant	2.495	0.707		3.53	0.001		
		Overall satisfaction	0.366	0.172	0.213	2.128	0.023	1	1

**Table 17:** Analysis of the relationship between satisfaction and behavioral intention of Xuhui Binjiang.

	Independent Variables		Non-Standard Coefficient		Standard Coefficients	*t*	Satisfaction Difference	Collinearity s = Statistics	
			B	Standard Error	Beta			Tolerance	VIF
Relationship between satisfaction of design elements and overall satisfaction of Xuhui Binjiang R^2^: 0.528, F value: 50.98 *p*: 0.000	Design elements	(Constant)	1.76	0.844		2.086	0.04		
		Site properties	0.271	0.134	0.212	2.021	0.046	0.871	1.148
		Natural environmental characteristics	−0.085	0.144	−0.065	−0.59	0.557	0.788	1.269
		Usage characteristics	0.367	0.182	0.222	2.021	0.046	0.799	1.252
		Administrative properties	0.087	0.137	0.064	0.637	0.526	0.963	1.038
Relationship between satisfaction of design elements and recommendation intention of Houtan Park (R^2^: 0378, F-value: 19.76, *p*-value: 0.218)	Design elements	(Constant)	2.999	1.263		2.375	0.02		
		Site properties	−0.139	0.201	−0.075	−0.691	0.492	0.871	1.148
		Natural environmental characteristics	0.255	0.216	0.136	1.18	0.241	0.788	1.269
		Usage characteristics	0.134	0.205	0.068	0.653	0.515	0.963	1.038
		Administrative properties	0.214	0.272	0.09	0.785	0.434	0.799	1.252
Relationship between satisfaction of design elements and revisit intention of Xuhui Binjiang Park R2: 0.730, F value: 52.29, *p*: 0.055	Design elements	(Constant)	2.463	1.424		1.729	0.087		
		Site properties	0.224	0.226	0.106	0.989	0.325	0.871	1.148
		Natural environmental characteristics	0.153	0.231	0.066	0.648	0.519	0.963	1.038
		Usage characteristics	0.593	0.243	0.274	2.438	0.017	0.788	1.269
		Administrative properties	0.087	0.307	0.232	2.283	0.186	0.799	1.252
Relationship between the overall satisfaction and revisit intention of Xuhui Binjiang Park R2: 0.508, F value: 66.48, *p*: 0.000	-	(Constant)	2.503	0.639		3.92	0		
		Overall satisfaction	0.497	0.161	0.300	3.08	0.003	1	1

## Data Availability

The data presented in this study are available on request from the corresponding author.
